# Automatic jargon identifier for scientists engaging with the public and science communication educators

**DOI:** 10.1371/journal.pone.0181742

**Published:** 2017-08-09

**Authors:** Tzipora Rakedzon, Elad Segev, Noam Chapnik, Roy Yosef, Ayelet Baram-Tsabari

**Affiliations:** 1 Faculty of Education in Science and Technology, Technion- Israel Institute of Technology, Haifa, Israel; 2 Department of Humanities and Arts, Technion- Israel Institute of Technology, Haifa, Israel; 3 Department of Applied Mathematics, Holon Institute of Technology, Holon, Israel; Institut Català de Paleoecologia Humana i Evolució Social (IPHES), SPAIN

## Abstract

Scientists are required to communicate science and research not only to other experts in the field, but also to scientists and experts from other fields, as well as to the public and policymakers. One fundamental suggestion when communicating with non-experts is to avoid professional jargon. However, because they are trained to speak with highly specialized language, avoiding jargon is difficult for scientists, and there is no standard to guide scientists in adjusting their messages. In this research project, we present the development and validation of the data produced by an up-to-date, scientist-friendly program for identifying jargon in popular written texts, based on a corpus of over 90 million words published in the BBC site during the years 2012–2015. The validation of results by the jargon identifier, the *De-jargonizer*, involved three mini studies: (1) comparison and correlation with existing frequency word lists in the literature; (2) a comparison with previous research on spoken language jargon use in TED transcripts of non-science lectures, TED transcripts of science lectures and transcripts of academic science lectures; and (3) a test of 5,000 pairs of published research abstracts and lay reader summaries describing the same article from the journals *PLOS Computational Biology* and *PLOS Genetics*. Validation procedures showed that the data classification of the De-jargonizer significantly correlates with existing frequency word lists, replicates similar jargon differences in previous studies on scientific versus general lectures, and identifies significant differences in jargon use between abstracts and lay summaries. As expected, more jargon was found in the academic abstracts than lay summaries; however, the percentage of jargon in the lay summaries exceeded the amount recommended for the public to understand the text. Thus, the De-jargonizer can help scientists identify problematic jargon when communicating science to non-experts, and be implemented by science communication instructors when evaluating the effectiveness and jargon use of participants in science communication workshops and programs.

## Introduction and objectives

Written communication is an essential part of advancing and understanding science [1]; accordingly, an integral part of learning and engaging in science is training to 'write' and 'talk' science. Communicating science can be divided into *scientific communication*, which refers to scientists sharing their work inside their community, and *science communication*, which refers to sharing science with non-experts [2]. In the traditional view “scientific communication can be represented as a continuum spanning from the research article, written for experts, to the popular science article, aimed at non-experts” (p. 26 [3]). One of the primary difficulties in adapting scientific texts from the academic to popular genre is the use of jargon, which is “the technical terminology or characteristic idiom of a special activity or group” [4]. Scientists are trained to speak in a highly specialized language. Communicating science without the use of jargon can become a challenge since scientists often suffer from what is known as the “curse of knowledge;” namely that people may not be able to remember that at one point they did not have the knowledge in question [5]. This may hamper scientists’ ability to communicate effectively with non-experts [6,7]. Scholars have stressed that to reach a non-expert audience, writing should try to avoid jargon, or gear jargon use to the audience [6,8–12]. But how can jargon be identified? Chung and Nation [13] suggested using computer and corpus work or a rating scale to judge technical terms.

This article details the development and validation of the data produced by an automated jargon online software identification program. The up-to-date, user-friendly on-line program was designed to help scientists and science communication educators to identify problematic vocabulary and adjust texts accordingly for purposes of public engagement with science.

Validation of the tool results took place in three stages: (a) comparing our results to another validated program, BNC-COCA/VPcompleat; (b) replicating results of jargon use from Sharon and Baram-Tsabari [11] based on TED talks and academic transcripts; and (c3) comparing jargon results from 5,000 pairs of published research abstracts and lay reader summaries describing the same article in *PLOS Computational Biology* and *PLOS Genetics* journals.

## Background

### The use of jargon in academic and popular science writing

Academic and popular science writing have contrasting characteristics. One can easily differentiate their vocabulary, goals, writing style, sentence structure, and internal structure [14]. Academic writing style is used in academic journals, conference proceedings and academic books. This style expects its readers to have previous knowledge of the field, familiarity with standard scientific article structure such as IMRAD (Introduction- Methods- Results- Discussion), and the use of academic vocabulary (e.g. analyze, facilitate), jargon (e.g. ion, cytokine). In contrast, popular science writing employs layperson terms and a narrative, journalistic style, and often draws on analogies and humor [12,15,16]. Popular science writing also stresses the uniqueness and novelty of research findings; it tends to remove hedges and qualifications and confer greater certainty on the findings [14].

One of the most fundamental differences between academic and popular science writing is vocabulary, and more specifically, technical jargon [3,11,12,14,17]. Jargon can alienate and exclude many audiences [18]. Plaxco [19] sees jargon as “one of the greatest enemies of clear scientific writing (p.2261),” and Turney [20] states “You never know what non-experts might know, but it is best to assume that they have little formal knowledge of the problems under discussion, little taste for the jargon of the field (p. 332).”

### Vocabulary lists and assessing reading comprehension

Previous studies have primarily employed the use of closed lists for general vocabulary, in which words were classified into different levels. Some vocabulary lists use *word types*, which refer to each word form individually, e.g., *value* and *values* are each unique word types, even though they belong to the same word family. A *word family* includes all the related word forms, e.g., *develop* would also include *undeveloped*, *underdeveloped*, *development*, *developments*, *developer*, and *developers*.

General vocabulary is often classified by frequency of use, as determined by evaluating written and spoken corpora, often from newspapers, magazines and books. General vocabulary has traditionally been divided into high frequency (1,000–3,000 word families) and low frequency (above 9,000-word family level). More recently, the literature has also presented a mid-frequency group (the 3000-9000-word family level) created from general vocabulary. Vocabulary level is a good predictor of reading comprehension [21,22], and has implications for learners of English as a second language [23]. Some general lists that are heavily used in the literature include the British National Corpus (BNC), comprising ~100 million words and the COCA [24] the largest free corpus of American English, including 520 million words. A combination of the BNC and COCA lists [25] has been divided into 25 levels. These lists were implemented in the validation process of the De-jargonizer tool output.

Research has also evaluated academic vocabulary, and has created academic lists from textbooks, academic book reviews, and master’s and doctoral theses [26,27]. These lists, primarily the New Academic Word List (AWL), include vocabulary for academic studies, excluding technical terms [28,29]. The New Academic Word list includes 570 word families created from a corpus of 3.5 million words of written academic texts, excluding the first 2,000 most frequently occurring word families in English [30]. The new AWL is estimated to cover 10% of all academic texts. Another academic list, the Academic Vocabulary List (AVL) [24] is a 120-million-word corpus from nine academic disciplines. AVL includes the top 570 word families, covering 14% of all academic texts.

Many studies have concentrated on creating field-specific vocabulary lists, for example in medicine [31,32], agriculture [33], chemistry [34], applied linguistics [35,36], engineering [37], and law [38]. These lists are aimed at helping pre-university English literacy and writing course instructors to prepare their students for higher education.

Overall, research using vocabulary lists has estimated that technical vocabulary (*e*.*g*. *polymerization*) typically makes up 5% of academic texts, whereas the bulk (80%) is made of high frequency words (*e*.*g*. *eye*, *animal*), and a smaller portion (8–10%) is composed of academic vocabulary (*e*.*g*. *derive*, *technique*) [21]. Hyland and Tse [27] found that academic science texts (specifically in the field of biology, physics, and computer science) had an even higher rate of technical vocabulary (22%). By comparison, Hu and Nation [39] found that a familiarity with 98% of all vocabulary (approximately the first 2,000 word families) in a text is required to accurately comprehend the content. In another study on vocabulary and comprehension in non-native adult readers, Laufer and Ravenhorst-Kalovski suggested a minimum level of comprehension, requiring knowledge of 95% of words in a text [23].

These considerations prompted us to generate an updated list that incorporates technical terms and determines their level of difficulty as a function of frequency. This fills the gap in current lists, which concentrate on frequency of general vocabulary while separately creating discipline specific lists. We place *common* technical jargon (e.g. *cancer*) into the high or mid-frequency list when appropriate, and determine what constitutes *rare* jargon (e.g. *chiral*) for lay audiences by its frequency in everyday news internet use.

### Assessing writing and jargon

Surprising few attempts have been made to categorize jargon, and even fewer to automatically identify it. One attempt to classify jargon and suggest which words should not be used in communicating science to lay audiences was published in Baram-Tsabari and Lewenstein [12]. They used *Google News* aggregator to classify words on a 5-point scale ranging from familiar (e.g. virus, galaxy) to jargon (e.g. meiosis, baryonic) based on the number of *Google news* hits. One disadvantage is *Google News* changes its algorithm and updates its corpus regularly, producing changing results for the same terms.

Sharon and Baram-Tsabari [11] used the British National Corpus (BNC) and Professional English Research Consortium (PERC) corpora to classify words from transcripts of TED design talks, science-related TED talks, and scientists’ academic lectures. The results indicated that scientists’ lectures directed toward the scientific community employed more jargon than lectures for TED design (non-science) talks or science-related TED talks. However, this work did not produce a tool that is readily implementable for teaching or self-assessment. Other online writing tools have been developed, such as prowritingaid.com [40], and grammarly.com [41]. These tools assess writing issues: they identify and correct grammatical, style and readability issues; they even recognize when words are overly repeated in a text; and they include a thesaurus to create synonyms if the reader chooses. However, they do not identify vocabulary level or jargon to rate a texts’ suitability for various audiences, and these tools provide only limited free services, charging a fee for full use of the program.

## Methodology

### Algorithm and design

The algorithm has been implemented in the website http://scienceandpublic.com/. Over 90 million words were tabulated in all the ~250,000 articles published on the BBC sites (including science related channels) from 2012 to2015. Overall, ~500,000 word types and names were ordered by number of appearances. These word types refer to unique words: for example, *disease* and *diseases* were each considered types, even though they belong to the same word family. The advantage of this system is that it can separate familiar from non-familiar types of words from the same family; for example, the familiar type *basis* and the less familiar type *basely*.

The size of our corpus is impressive: the 2002 Webster's Third New International Dictionary evaluated the contemporary American lexicon at approximately 348,000 single-word forms [42], and Michel et al. [43] estimated there are 1,022,000 individual word forms in the English lexicon in a computer analysis of a corpus comprising over 5 million digitized books. We chose the BBC as an up-to-date representation of general and general-science related vocabulary. To correct for differences in American spellings of words in the corpus, we added American spellings of words as well [44].

All the articles in the corpus were crawled online using the scrappy framework (http://scrapy.org/) and all data was uploaded to figshare open source archive, which includes original site files and information about the site, database construction, and technologies used. Crawling only included editorial content. Advertisements, reader comments, phone numbers, website URLs, and email addresses were ignored. In addition, punctuation was ignored, hyphenated words were separated, and apostrophes were deleted (example: ‘s). Single new words were extracted to an Excel sheet, and the number of appearances in the corpus was recorded.

**Fig 1:**
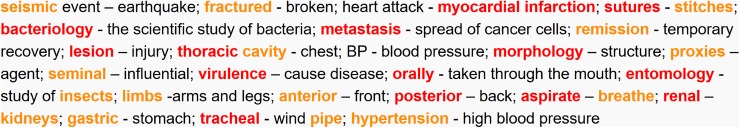
Screen shot showing sample output from the De-jargonizer. Jargon terms in red (58%), and their lay explanations in black (common/high frequency 21%) and orange (mid-frequency, 21%).

The words in the text were then classified to one of three levels based on the following cutoffs: high frequency (e.g. *behavior*), mid-frequency (e.g. *protein*) and jargon (e.g. *dendritic*). Since the threshold between high frequency and mid-low frequency words is best made after the 2000 word family level [45], we used this as a guideline. High frequency words were color coded in black representing the most common words, akin to the 2000 most frequent word families. These appeared in over 1000 uses in the corpus. Mid-frequency words were color coded in orange. This level contains words which should be familiar to intermediate and advanced readers [45]. They appeared fewer than 1,000 but more than 50 times in the corpus. Jargon was color-coded in red, and represented words that are most likely unfamiliar to non-experts, and appeared fewer than 50 times in the corpus. This color coding of the text allows the reader to spot the jargon (Fig 1).

The frequency of each vocabulary level, presented by number of words from each frequency and their percentage out of the total words in the text, is shown in the Fig 1 caption, and in a block next to the text when using the site. These frequencies break down the text for lay audiences; however, users can interpret their own results for various reading levels using the guide on the site (http://scienceandpublic.com/Home/Instructions#judgingResults). In addition, a total score was defined as 0–100, such that if all the words in the text are common, the text score is 100; each mid-frequency or jargon word reduces the score based on the following equation:

$$score=100-\frac{1}{2}\left(\frac{number\;of\;midfreq.\;words}{total\;words\;in\;the\;text}\right)-\left(\frac{number\;of\;Rare\;words}{total\;words\;in\;the\;text}\right)$$

An additional advantage of the De-jargonizer site is that its word list and frequencies will be updated yearly. Moreover, the site is flexible and analyzes various file formats, including txt, htm, html, and docx files.

### Development

**Fig 2:**
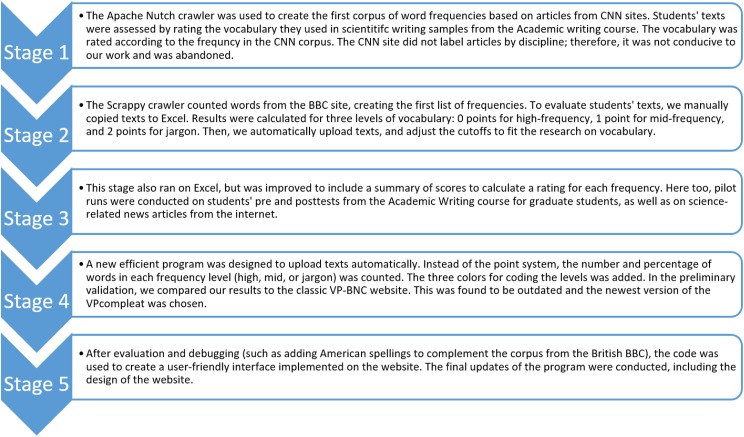
The De-jargonizer: Five stages of testing and development. All stages included trial runs on student writing samples.

Overall, the system for jargon identification was in development for 3 years. Five pilot versions and platforms were tested and improved until the frequency lists, categories, and online interface produced valid output in the final stage (Fig 2). Each round included an assessment of authentic STEM graduate students' texts in a Graduate Academic Writing course taught by one of the authors. The course is a 14-week long compulsory course for Ph.D. students at a technological university. Students enrolled in the course took an identical pre- and post-assessment test at the beginning and end of the semester. The test asked students to “*Describe your research*, *its context and implications in English for (A) a general audience (no science background) and (B) the academic community*, *in 150–250 words each*” (see full syllabus, interventions and results of the implementation in [17]). These were used to analyze the utility and ease of the program, as well as to measure actual students’ outcomes across writing interventions and coursework.

## Validation

Few studies present the validation process for data produced by language instruments; however, those that do concur that “assessment tools need to be valid (i.e. measure what they purport to measure) and reliable (i.e. measurements need to be free from measurement error as much as possible)” [46]; moreover, “Score validity deals with the degree to which scores from a measurement measure the intended construct (p. 5)” [47].

Therefore, our validation stages included a comparison to existing measures in the literature [11,25] to achieve convergent validity; i.e., "the evaluation of how well an index agrees with other indices that are widely accepted as a standard against which to measure a given construct [48,49]. Below are the three validation stages.

1. *Comparison to an established word frequency list*. Results from the De-jargonizer software were compared to results from the BNC-COCA VPcompleat program [25,50]. Pre/post academic texts and popular writing samples from ~250 students at an academic writing course were used. T-tests were used to compare pre-post change in jargon use. To control for length, scores were divided by the number of words.
2. *Jargon in popular and academic science transcripts*. A comparison of the results was made with the evaluator in Sharon and Baram-Tsabari [11] which identified jargon in *28* transcripts of TED design (non-science) lectures, 31 TED science lectures and 43 advanced undergraduate level science and engineering transcripts from the Michigan Corpus of Academic Spoken English [51]. ANOVAs were used to compare the jargon identifier results across the three groups. These results were compared to the findings in Sharon and Baram-Tsabari [11].
3. *Jargon in pairs of abstracts and lay summaries*. A comparison was made of the language between academic abstracts and lay summaries in *PLOS* research articles. The instructions for the academic abstract and author summary can be found on the PLOS site at: http://journals.plos.org/ploscompbiol/s/submission-guidelines. Whereas academic abstract instructions are similar to guidelines in many scientific publications, the instructions for summaries are as follows (bold added for emphasis):

“We ask that all authors of research articles include a 150–200-word non-technical summary of the work …. **avoid the use of acronyms and complex terminology wherever possible. The goal is to make your findings accessible to a wide audience that includes both scientists and non-scientists.** Authors may benefit from consulting with a science writer or press officer to **ensure they effectively communicate their findings to a general audience**.”

Kuehne and Olden [52] argued that summaries for the lay audience, such as those used in *PLOS Biology*, *PLOS Neglected Tropical Diseases*, *PLOS Genetics*, *PNAS*, *Behavioral Ecology*, *Functional Ecology*, *Frontiers in Ecology and the Environment*, among other journals can contribute greatly to science communication by providing an opportunity for science communication with the public, more transparency and accessibility to science among laypersons and policy makers, and control over the content to avoid misrepresentation.

Published research abstracts and lay reader summaries describing the same article (n = 5000 pairs) from *PLOS Computational Biology* and *PLOS Genetics* journals were tested in the De-jargonizer program (an example of a tested abstract and the corresponding lay summary from *PLOS Computational Biology* can be found in the ‘Results’). T-tests were used to compare the results.

## Findings

The results from each of the three stages of validation are presented: comparison to an established word frequency list, jargon in popular and academic science transcripts, and jargon in pairs of abstracts and lay summaries. Overall, the De-jargonizer exhibited good agreement when replicating previous results [11,25], and adequately identified jargon levels in academic abstracts as compared to lay summaries. Moreover, through the analysis of lay and academic texts, the De-jargonizer revealed that many lay texts remain far beyond the recommended level of vocabulary.

### Comparison to an established classifier

Four types of student writing samples were run though the BNC-COCA VPcompleat program and the De-jargonizer. The samples comprised academic and popular posttests of an academic writing course (~30,000 words for each genre) composed by 76 STEM graduate students. Results were compared for correlations to assess validation of the scores of the new jargon software and identify unique features of our program. The results showed that the De-jargonizer’s high frequency word level correlates with the first most frequent 2000-word families (i.e. levels K1+K2) in the BNC-COCA programs for academic (*r* (76) = .807, p ≤0.01) and popular science texts (*r* = (76) = .795, p ≤ 0.01). In addition, words that the De-jargonizer classified as jargon were comparable to the words beyond the BNC-COCA 6000-word family level (K6- K25 and offlist) for academic (*r* (76) = .808, p ≤ 0.01) and popular science texts (*r* (76) = .757, p ≤ 0.01).

**Fig 3:**
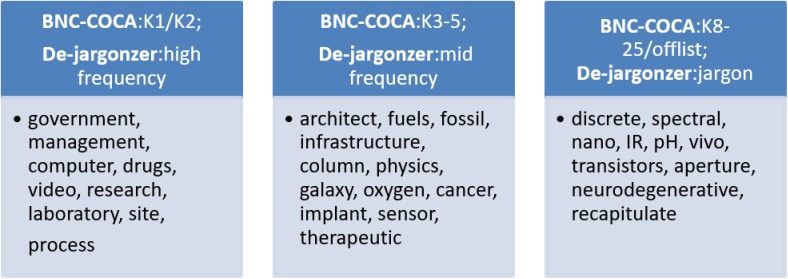
Examples of words that were classified similarly by the De-jargonizer and the BNC-COCA. Each column shows the levels and the corresponding words found to be classified similarly. Note: levels K1+K2 are first most frequent 2,000 word families according to BNC-COCA; each K = 1,000 word families up to K25, followed by offlist words beyond K25.

Fig 3 shows several examples of jargon that match identifications in both programs, including words such as *government*, *computer* and *drugs* as high level/K1-2 frequencies. In contrast, the De-jargonizer differs in classifying up-to-date words and terms. For example, *functionally* is classified as K3, but as jargon in our system; widely known words such as *Google*, *worldwide*, and *PhD*, as well as names of diseases that are part of everyday language such as *Alzheimer’s* and *Parkinson’s*, are classified as high frequency in the De-jargonizer, while they are offlist in the BNC program.

### Jargon in popular and academic transcripts

ANOVAs showed a main effect of (*F* (2, 104) = 70.3) in use of jargon between the three lecture groups: TED design, TED science, and scientific lectures. Posthoc analyses using Tukey’s HSD indicated that the three lecture groups had significant differences (p ≤ 0.05) in use of jargon: significantly higher jargon was used by the TED design group compared to the academic scientific lectures group, and by the TED science group compared to the academic scientific lectures group. No significant difference in jargon prevalence between TED design and TED science was found. Sharon and Baram-Tsabari [11] found similar results; i.e., academic scientific lectures contained significantly more jargon than TED design or science lectures.

### Comparison of abstracts and lay reader summaries

Differences in vocabulary use between academic abstracts and general audience summaries in published research articles in *PLOS* journals were tested using t-tests (n = 5000). Results showed academic abstracts in *PLOS Computational Biology* employed an average of 10% (SD = 5%) jargon and lay summaries used 8% (SD = 4%). *PLOS Genetics* academic abstracts employed an average of 17% jargon (SD = 6%), and lay summaries used no less than 12% jargon words (SD 6%).

**Fig 4:**
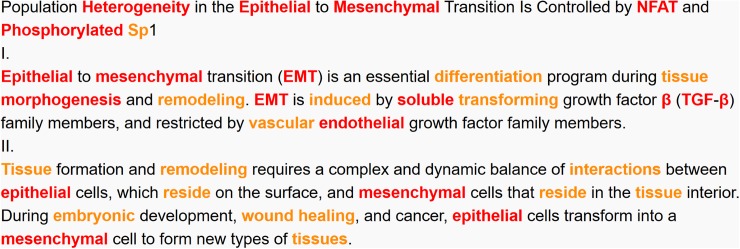
Example comparison of an excerpt of an abstract and corresponding lay summary. Part I (abstract) has 57% high frequency 16% mid-frequency and 27% jargon. Part II (summary) uses 71% high frequency, 20% mid-frequency, and 8% jargon. Excerpts taken from [53].

Indeed, there was a significant difference in the use of jargon between the abstract and summary; i.e., more jargon was found in the academic abstract (M = 26.62; SD = 14) (p = .000). However, the percentages of jargon in the lay summaries were still far beyond the recommended 2% jargon word coverage [39] in both journals at 8% and 12% respectively. Fig 4 presents a sample excerpt of a color-coded abstract with 27% jargon, and the corresponding lay summary with 8% jargon.

## Discussion

This project developed and validated the data produced by an automated jargon identification program, the De-jargonizer. The program can help scientists and science communication instructors improve and adapt vocabulary use when communicating with non-experts. The program determines the level of vocabulary and terms in a text, and divides the words into three color-coded levels: high frequency/common words, mid-frequency words, and jargon/rare words. The De-jargonizer highlights jargon, thus allowing science speakers and writers to consider changing these potentially problematic words for more familiar words or adding explanations.

The findings exhibit good fit with all three stages of our validation of the jargon identifier. In testing our system against the validated BNC-COCA program [25], high correlations support that the program generates valid data. Two key practical advantages of the De-jargonizer emerge from this comparison. The system allows for a simple 3-tiered rating system that is color coded to easily spot difficult terminology and allows for quick and easy identification of jargon for scientists. In addition, the De-jargonizer includes an up-to-date classification of words, since it does not use a close-ended corpus of texts, but can be easily updated with new texts and data sources tailored for specific audiences. It also allows scientists and science communication educators to evaluate the percentage of jargon in a text.

The comparison of jargon use in popular and academic transcripts to findings in [11] also found good agreement. Both TED design and TED science lectures had significantly less jargon than academic scientific lectures, supporting the use of our program in identifying jargon in oral transcripts, and obviating the need to analyze the texts with several different programs.

In the third stage, we compared corresponding academic abstracts and lay summaries in research articles in *PLOS* academic journals. Lay summaries showed significantly less use of jargon than academic abstracts. However, the percentages of jargon in the lay summaries hugely exceeded the recommended 98% vocabulary familiarity level [23,39]. Our program can help identify and rectify this issue, by guiding journal writers to meet certain predetermined text levels. It may assist scientists in writing lay summaries, press releases and opinions for the popular media, as suggested by [52,54–56], allowing an increasing amount of research to be accessible by the public.

Limitations of the program include cases in which the same word holds a different meaning for scientists and layperson. For example, the jargon identifier marked *fatigue* as mid frequency (and in the K5 BNC-COCA) but a closer look at the text showed that it referred to the technical meaning of the word in material and mechanical engineering. Further research could include a list of terms that differ from lay to professional audiences and at least mark them for further consideration (e.g. "positive feedback"). In addition, future research should investigate actual reader’s comprehension of popularized scientific texts with varying jargon levels. In the future, the system could be modified to offer alternative words to replace jargon, and be adapted to measure vocabulary for different audiences at various levels.

## Social and educational implications

The importance of making science and research accessible to the public is clear in today’s world. Science communication helps create more informed citizens, and according to a recent editorial in Nature: “scientists who engage using social media might productively influence the public discourse” [57]. While written texts are only part of the venues of science communication, they are a central venue by which many people receive their news and shape their ideas about science [58].

Our results show that when journals include both an academic abstract and a lay summary, scientists are trying to adapt their language and ideas to a wider public. However, our work also points out that even when considering the basic criteria of vocabulary, many summaries require more adaptation to reach the ideal percentage of unfamiliar words [23,39]. This highlights the need for science communication training as a form of professional development and as part of graduate level education. Science communication educators can use the program to assess the progress of their students by evaluating their choice of appropriate vocabulary, and compare the effectiveness of different interventions [59–61].

Specific professional fields, such as medicine, could use such an updating tool as well to evaluate text level and its suitability to lay audiences. Research has shown that patients sometimes cannot understand doctors’ explanations and instructions, leading to a host of calls encouraging doctors to avoid jargon [62–66]. Such a tool may aid doctors in adapting their communications. A variety of experts who communicate outside their field, such as lawyers and economists, may also find it useful. A US law, the Plain Writing Act of 2010 [67], further supports the avoidance of jargon, requiring “the effectiveness and accountability of Federal agencies to the public by promoting clear Government communication that the public can understand and use”. The De-jargonizer provides an up-to-date and user-friendly tool to improve these communications by analyzing one's text, and allowing communicators to adapt it to lay audience level and fight professional norms and curse of knowledge [5] to make expert-public communication more effective.
